# Impaired smooth-pursuit in Parkinson's disease: normal cue-information memory, but dysfunction of extra-retinal mechanisms for pursuit preparation and execution

**DOI:** 10.14814/phy2.12361

**Published:** 2015-03-29

**Authors:** Kikuro Fukushima, Norie Ito, Graham R Barnes, Sachiyo Onishi, Nobuyoshi Kobayashi, Hidetoshi Takei, Peter M Olley, Susumu Chiba, Kiyoharu Inoue, Tateo Warabi

**Affiliations:** 1 Department of Neurology, Sapporo Yamanoue HospitalSapporo, Japan; 2 Faculty of Life Sciences, University of ManchesterManchester, UK; 3 Department of Radiology, Sapporo Yamanoue HospitalSapporo, Japan

**Keywords:** Extra-retinal mechanisms, memory, Parkinson's disease, priming effects, smooth-pursuit

## Abstract

While retinal image motion is the primary input for smooth-pursuit, its efficiency depends on cognitive processes including prediction. Reports are conflicting on impaired prediction during pursuit in Parkinson's disease. By separating two major components of prediction (image motion direction memory and movement preparation) using a memory-based pursuit task, and by comparing tracking eye movements with those during a simple ramp-pursuit task that did not require visual memory, we examined smooth-pursuit in 25 patients with Parkinson's disease and compared the results with 14 age-matched controls. In the memory-based pursuit task, cue 1 indicated visual motion direction, whereas cue 2 instructed the subjects to prepare to pursue or not to pursue. Based on the cue-information memory, subjects were asked to pursue the correct spot from two oppositely moving spots or not to pursue. In 24/25 patients, the cue-information memory was normal, but movement preparation and execution were impaired. Specifically, unlike controls, most of the patients (18/24 = 75%) lacked initial pursuit during the memory task and started tracking the correct spot by saccades. Conversely, during simple ramp-pursuit, most patients (83%) exhibited initial pursuit. Popping-out of the correct spot motion during memory-based pursuit was ineffective for enhancing initial pursuit. The results were similar irrespective of levodopa/dopamine agonist medication. Our results indicate that the extra-retinal mechanisms of most patients are dysfunctional in initiating memory-based (not simple ramp) pursuit. A dysfunctional pursuit loop between frontal eye fields (FEF) and basal ganglia may contribute to the impairment of extra-retinal mechanisms, resulting in deficient pursuit commands from the FEF to brainstem.

## Introduction

Idiopathic Parkinson's disease is a progressive neurodegenerative disorder characterized by loss of dopaminergic neurons in the substantia nigra pars compacta of the basal ganglia. Most patients with Parkinson's disease suffer from somatomotor and oculomotor disorders (see Rowland and Pedley 2010 for review). The oculomotor system facilitates obtaining accurate information from the visual world by keeping the fovea on an object of interest. If a small object of interest moves slowly in the frontoparallel plane, tracking eye movements occur that consist primarily of smooth-pursuit interspersed with correction saccades (see Leigh and Zee 2006 for review).

Although impaired pursuit in Parkinson's disease is well known, reports are conflicting. Using sinusoidal target motion, early studies reported a low gain (peak pursuit eye velocity/peak target velocity), resulting in “saccadic pursuit” (Shibasaki et al. 1979; White et al. 1983; Gibson et al. 1987; Sharpe et al. 1987; Vidailhet et al. 1994; Rottach et al. 1996; Lekwuwa et al. 1999; Bareš et al. 2003; Pinkhardt et al. 2008, 2009, 2012), although Rascol et al. (1989) reported that pursuit velocity was normal at a low frequency (0.2 Hz), but reduced at a higher frequency (0.4 Hz). Rottach et al. (1996) reported latency prolongation with low pursuit acceleration during step-ramp stimuli (also Shibasaki et al. 1979). However, it is well known that normal aging alone results in changes in pursuit responses such as low gain/pursuit eye velocity and latency prolongation (e.g., Moschner and Baloh 1994; see Leigh and Zee 2006 for review). These changes could be explained simply by age-related changes in neuronal activity in the cortical visual/pursuit pathways without including the basal ganglia (e.g., Leventhal et al. 2003; see Discussion of Fukushima et al. 2014). To understand the specific nature of impaired pursuit in Parkinson's disease requires a more in-depth understanding of how the smooth-pursuit system operates.

While visual motion on the retina is the primary input for smooth-pursuit, its latency is much shorter than latencies of saccades or manual movements to visual stimuli in normal subjects, and its efficiency for short-latency smooth tracking (vs. “saccadic tracking”) depends on cognitive processes (for reviews, see Leigh and Zee 2006; Barnes 2008; Fukushima et al. 2013). Priming contributes to shorter latencies. For example, initial slow eye movement responses induced by visual motion inputs arising from two identical spots moving in opposite directions with the same speed are known to be nullified due to vector averaging in both monkeys and humans, resulting in no initial pursuit (Lisberger and Ferrera 1997; Garbutt and Lisberger 2006); subjects start tracking eye movements with saccades, and smooth-pursuit appears after saccades. Priming by preceding cue-information shortens saccade latencies and induces initial pursuit before the saccades in the cued direction (Bichot and Schall 2002; Garbutt and Lisberger 2006; Fukushima et al. 2008, 2011c, 2013). Furthermore, Barnes and Collins (2008a,b) have shown that normal humans can initiate smooth-pursuit by anticipation even when the target is extinguished shortly before motion onset, indicating predictive pursuit initiation by extra-retinal drive (also Helmchen et al. 2012).

In studies using predictive or nonpredictive spot motion stimuli, impaired prediction is reported in Parkinson's disease (Ladda et al. 2008; Helmchen et al. 2012), although preserved predictive function was also reported (Flowers and Downing 1978; Bloxham et al. 1984; Bronstein and Kennard 1985; Waterston et al. 1996; Lekwuwa et al. 1999; Pinkhardt et al. 2009). Helmchen et al. (2012) reported that patients with Parkinson's disease had difficulties in initiating pursuit when the target was extinguished shortly before motion onset but pursuit maintenance was preserved when the target was blanked during pursuit, suggesting impaired extra-retinal drive.

We conjectured that the inconsistent results in patients with Parkinson's disease may partly reflect differences in prediction-related cognitive effects on their tracking performance, since prediction could occur not only in motor commands to prepare for or maintain ongoing movements, but also in sensory/perception processes such as target motion memory (e.g., Becker and Fuchs 1985; cf., Barborica and Ferrera 2003). Moreover, in daily life, there are often multiple moving objects, which require selection of a specific target, and include deciding whether or not to, and what to pursue. To separate these prediction-related components, a cue-dependent memory-based pursuit task has been used in trained monkeys (Fukushima et al. 2008, 2011a; Shichinohe et al. 2009) and normal human subjects (Ito et al. 2013a; Fukushima et al. 2014).

By comparing tracking eye movements of both primate species during the memory-based pursuit task and a simple ramp-pursuit task that did not require visual memory, we showed that tracking eye movements of the two subject groups were similar in each task but their tracking eye movements were different between the two tasks (Ito et al. 2013a). We identified important extra-retinal mechanisms for initiating pursuit, including cue-information priming and extra-retinal drive (Ito et al. 2013a; also Barnes and Collins 2008a,b). Further comparison of young and elderly human subjects indicates normal aging affects movement execution including pursuit latencies, velocities, and accelerations, but not visual memory nor appearance of initial pursuit induced by the extra-retinal mechanisms (Sprenger et al. 2011; Fukushima et al. 2014). Moreover, the difference in pursuit latencies between the two tasks, that includes decision-making delay in the memory task, was similar between young and elderly subjects, indicating that these functions are little affected by normal aging (Fukushima et al. 2014).

These observations suggest that they may well provide specific information on impaired pursuit in Parkinson's disease. Previous studies reported impaired working memory during cognitive tasks in Parkinson's disease (Possin et al. 2008; Lee et al. 2010; cf., Ladda et al. 2008). As studies using trained monkeys indicate different cerebral areas carry distinctly different signals during memory-based pursuit, and because selective chemical inactivation of some of these areas induces specific expected effects (Shichinohe et al. 2009; Fukushima et al. 2011a; also Kurkin et al. 2011, 2014), understanding specific nature of impaired smooth-pursuit in Parkinson's disease may well provide insight on possible pathophysiology for the impaired pursuit in this disease.

Our objective in this study was to clarify the specific nature of impaired pursuit in Parkinson's disease in the following aspects and to deduce pathophysiology of this disease; (1) cue-information memory during memory-based pursuit, and (2) the extra-retinal mechanisms for pursuit initiation based on the cue-information memory. We compared tracking eye movements of patients with Parkinson's disease and age-matched control subjects during the three task conditions used previously in normal subjects (Ito et al. 2013a; Fukushima et al. 2014). Some of the results were presented in preliminary form (Fukushima et al. 2011b,c, 2013; Ito et al. 2011, 2012, 2013b).

## Materials and Methods

### Subjects

Subjects were 30 patients with Parkinson's disease aged 56–87 and 14 age-matched normal subjects aged 54–89 as controls. Diagnosis of Parkinson's disease was based on the UK Parkinson's Disease Society Brain Bank clinical diagnostic criteria (Hughes et al. 1992). All subjects were recruited at Sapporo Yamanoue Hospital. This study complied with the Declaration of Helsinki. The Sapporo Yamanoue Hospital Ethics Committee approved the specific procedures. Each subject was informed for this study and the procedures involved prior to giving their consent.

Table1 summarizes clinical characteristics. Of the 30, five patients had difficulty in understanding the memory-based pursuit task (see below for details) and they declined to perform the task (Table1, Pt #26–30), although they had no difficulty in performing the simple ramp-pursuit task (see below). The remaining 25 patients performed our tasks (Table1, Pt #1–25, 56–87 years). They had mild tremor, rigidity, postural/gait disturbances with mean Hoehn and Yahr (1967) stage of 2.9 ± 0.6 SD. In 29 of the 30 patients, we also evaluated the unified Parkinson's disease rating scale (UPDRS part 3, Fahn and Elton 1987) as summarized in Table1. Of the 25 patients who performed our tasks, six were untreated by anti-parkinsonian medication (e.g., levodopa/dopamine agonists) prior to this study (Table1, Medication, No drug).

**Table 1:** Clinical characteristics. Patient (Pt) # 1–30. Age in years. Medication indicates anti-parkinsonian medication

Pt No.	Age/sex	H-Y stage	UPDRS part 3	Duration	Main clinical symptoms	Brain MRI	MMSE	FAB	Medication, daily dose (mg)	Initial Pursuit	
										MP	SR
1	56/F	3	11	3 years	Lt. resting tremor	Normal	29	14	No drug	−	+
2	56/M	3	16	7 years	Bradykinesia, Lt. rigidity, REM sleep disorder	Bil. BG lacunar infarct.	29	18	No drug	+	+
3	57/F	1	14	1.5 years	Lt. rigidity, resting tremor	Normal	30	17	No drug	+	+
4	59/F	3	3	4.5 years	Gait festination, freezing Lt. rigidity	Normal	26	16	LeBe 200/50, En 200, Pr 2.5	−	−
5	64/F	3	28	4 years	Lt. rigidity, resting tremor	Normal	30	14	LeBe 150/37.5, Pr 0.75	−	+
6	65/M	3	21	1 year	Gait festination	Normal	25	15	No drug	+	+
7	66/M	3	17	6 years	Gait freezing, Lt. resting tremor, rigidity	Lt. arachnoid cyst	27	15	LeCa 200/20, Pr 2, En 300, Dr 300	−	+
8	70/F	3	36	3 years	Rt. rigidity, resting tremor	Normal	26	16	LeCa 300/30, Pr 2.5	+	+
9	70/F	3	24	11 years	Flexed posture to rt.	Normal	23	14	LeCa 450/112.5,Tri 4, Se 5, Per 1.125, En 400	+	+
10	72/M	2	16	2 years	Bradykinesia	Normal	30	17	LeBe 300/75	+	+
11	73/F	2	23	1 year	Rt. rigidity Gait festination	Normal	28	15	No drug	−	+
12	74/F	2.5	13	4 years	Lt. rigidity	Normal	28	15	LeBe 150/37.5, Pr 0.5	−	+
13	75/F	3.5	45	2 years	Rt. resting tremor, Forward flexed posture	Normal	24	16	Ro 4	−	+
14	75/M	3	Not tested	6.5 years	Bradykinesia	Not tested	26	15	LeCa 600/60, Pr 2	−	−
15	77/F	3	31	9 years	Gait freezing	Normal	30	18	LeBe 300/75, Pr 2.5 Am 250, Se 7.5, Dr 300	−	Not tested
16	77/F	3	29	7 years	Flexed posture to rt., Rt. resting tremor, Dyskinesia	Normal	26	16	LeBe 300/75, Pr 2, En 300, Am 50	−	−
17	77/M	3	13	4 years	Gait freezing, Bil. rigidity	Normal	29	15	LeBe 300/75, Am 250, Se 7.5, Dr 300	−	−
18	78/F	3	23	1 year	Rt. resting tremor	Bil. BG lacunar infarct.	25	15	LeCa 300/30, Pr 1.5	−	+
19	79/M	3	30	1 year	Gait festination, Lt. rigidity	Normal	28	14	LeBe 200/50, Pr 1, Am 100	−	+
20	80/M	4	32	22 years	Rt. resting tremor	Bil. BG lacunar infarct.	30	15	LeCa 350/35, Ro 9, Am 100, Se 7.5, Dr 600, En 300, Donepezile 5	−	+
21	83/M	3.5	24	5 years	Gait festination, freezing, Rt. resting tremor, rigidity	Normal	29	13	LeBe 400/100, Pr 2, En 400	−	+
22	84/M	3.5	53	2 years	Gait festination	Normal	24	14	LeCa400/40	−	+
23	86/M	3.5	29	4 years	Gait festination, Hallucination	Small bleeding in Rt. putamen	21	11	Pr 0.125	−	+
24	87/F	2	28	2 m	Gait festination, Rt. rigidity, resting tremor	Bil. BG lacunar infarct.	20	12	No drug	−	+
25	80/F	3	31	5 years	Lt. resting tremor	Normal	25	10	Ro 0.25, LeCa 200/20, Dr 300	−	−
26	71/F	3	20	1 year	Gait festination	Lacunar infarct. around bil. lateral ventricles	24	13	No drug	Not tested	+
27	78/F	2.5	13	2 m	Forward flexed posture, Gait dfestination	Normal	23	11	No drug	Not tested	+
28	77/F	3.5	31	2 years	Bil. resting tremor, Bradykinesia	Mild atrophy of cerebral cortex	22	8	LeBe 400/100	Not tested	+
29	78/M	3.5	30	9 years	Lt. resting tremor, Rt. hand rigidity	Normal	25	12	LeBe 500/125, Rp 1, Dr 200, En 600	Not tested	−
30	80/M	3	28	11 years	Gait freezing	Normal	28	7	Am 150, En 300, LeCa 300/30, Dr 400	Not tested	−

No drug indicates that patients received no anti-parkinsonian medication prior to this study. + and − in Initial pursuit indicate presence and absence, respectively, of initial pursuit during memory-based pursuit (MP) and simple ramp-pursuit (SR).

F, female; M, male; H-Y, Hoehn–Yahr; y and m, disease duration in years (y) or months (m); Lt. left. Rt; right. BG, basal ganglia; Bil, bilateral; REM, rapid eye movement; MMSE, mini-mental state examination; FAB, frontal assessment battery; MP, memory-based pursuit; SR, simple ramp-pursuit; UPDRS, unified Parkinson's disease rating scale; Am, amantadine; Dr, droxidopa; En, entacapone; LeBe, levodopa-benserazide; LeCa, levodopa-carbidopa; Per, Pergolide mesilate; Pr, pramipexole; Ro, ropinirole; Se, selegiline; Tri, trihexyphenidyl hydrochloride.

All patients performed the mini-mental state examination and frontal assessment battery (Table1, MMSE, FAB; Folstein et al. 1975; Dubois et al. 2000). Mean ± SD scores of the 25 patients for MMSE and FAB were 26.7 ± 2.9 and 14.8 ± 1.9, respectively, whereas those of the five patients who declined to perform our memory task were 24.4 ± 2.3 and 10.2 ± 2.6. Although MMSE scores were similar in the two groups of patients (median 27 and 24, respectively, *P* = 0.07, Wilcoxon–Mann–Whitney Test), FAB scores were lower in the latter group (median 15 and 11, *P* = 0.002), especially in conceptualization, mental flexibility, and motor programming scores (Dubois et al. 2000), suggesting their difficulty in these functions. UPDRS scores of the two groups were similar with mean ± SD of 24.6 ± 11.1 (Table1, Pt #1–25) and 24.4 ± 7.7 (Pt #26–30) (median 24 and 28, respectively, *P* = 0.954, Wilcoxon–Mann–Whitney Test). Main clinical symptoms and brain MRI findings were basically similar between the two groups (Table1).

All 14 normal subjects had no history of any condition likely to affect eye movement, and were not on any medication known to affect the oculomotor system. Of the 14, three were family members of the Parkinson's disease patients; five were medical doctors; and the remaining six were outpatients of other departments at Sapporo Yamanoue Hospital. These six subjects performed MMSE and three of the six performed FAB. Mean±SD MMSE and FAB scores were 26.2 ± 2.0 and 11.7 ± 1.5, respectively, comparable to the values of the 25 patients (Table1, Pt #1–25).

### Eye movement recordings and memory-based pursuit task

**Figure 1:**
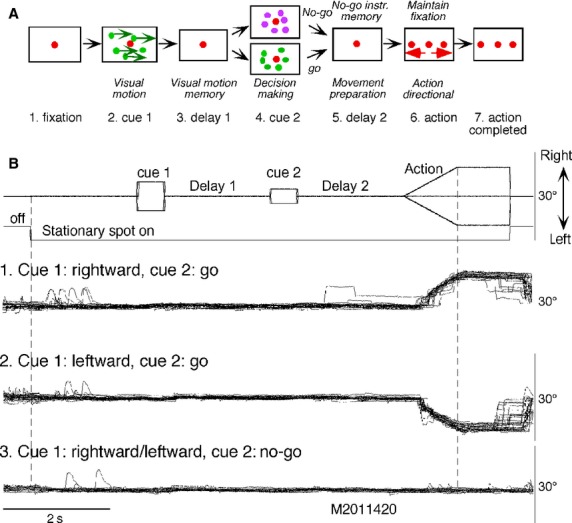
Task conditions and eye movements of a patient with Parkinson's disease aged 74. (A) memory-based pursuit task conditions. (B1–3) sorted eye position traces to cue 1/cue 2 directions/instructions as indicated. See text for further explanation.

Details of stimulus presentation and data analysis were described previously (Ito et al. 2013a). An infrared limbus tracking device (d.c. −100 Hz, −24 dB/octave, Takei, Japan) was used to record horizontal movements of the right eye. Subjects sat with their head immobilized by a chin rest and a forehead restraint. A monitor screen (22 inch, 120 Hz) was positioned 70 cm in front of the subject's eyes under room lighting. The subjects were asked to fixate a 1° stationary spot at the screen center until the action period (Fig.1A6). After 2 sec initial fixation (Fig.1A1), cue 1 appeared, which consisted of a circular random-dot pattern of 10° diameter. Each dot in the pattern moved either rightward or leftward at 10°/s for 0.5 sec (Fig.1A2). Subjects were asked to remember the pattern color and the movement direction. After a 2 sec delay (Fig.1A3), a similar, but stationary, circular random-dot pattern was presented as the second cue for 0.5 sec (Fig.1A4). If the color of cue 2 dots was the same as the cue 1 color, the subjects were instructed to prepare to pursue a spot that would move in the direction instructed by cue 1 (i.e., go). If the cue 2 color was different from cue 1, it instructed the subjects not to pursue (i.e., no-go) but to maintain fixation of a stationary spot (Fig.1A6).

We prepared five sets of different-colored random dots for cue 1 and cue 2, and presented each set randomly within a block of trials. After 2 sec delay (Fig. 1A5), the stationary spot remained, but spawned two identical spots; one that moved in the direction instructed by cue 1 and the other moved in the opposite direction at 10°/s for 1 sec (Fig.1A6). The subjects were asked to execute the correct action by selecting one of three spots and either pursuing the correct spot in the correct direction (i.e., go) or not to pursue (i.e., no-go) by maintaining fixation of the spot that remained stationary (Fig.1A6). After the action period, the three spots remained stationary at each location for 1 sec (Fig.1A7) followed by a blank screen for 1.5 sec to give subjects time to blink. It took about 10s for one trial, and one block typically consisted of 30 trials. Two blocks were typically tested. The frequency of occurrence of fixation (i.e., no-go) trials was set at 24%, and in the remaining 76% of the trials the subjects were required to pursue one of the two moving spots (i.e., go) as described above.

We asked each subject to participate in a few practice trials while giving verbal feedback to be sure that they understood the task. Occasionally, during delay 2 of go trials, some patients with Parkinson's disease made saccades toward the correct direction before the onset of action period (e.g., Fig.1B1). After asking them to follow the correct spot motion but not to make saccades before its motion, their performance was recorded. Whenever subjects showed signs of fatigue, trials were stopped for short periods (∽30 sec).

### Simple ramp-pursuit task

To estimate the effects of visual memory on tracking eye movements during go trials, we examined eye movements during simple ramp-pursuit that were initiated primarily by retinal image motion and did not require visual motion direction memory. A 1° single spot was used that moved with the identical motion trajectory. This spot stayed stationary at the screen center for 2 sec, similar to the initial fixation of the memory-based pursuit task (Fig.1A1), and then moved either rightward or leftward randomly at 10°/s for 1 sec. The spot remained stationary at 10° either right or left for 1s followed by a blank period of 1.5 sec. Subjects were asked to fixate the spot when it appeared and to follow its movement. One patient with Parkinson's disease declined to perform this task due to fatigue (Table1, Pt. #15, SR, not tested).

### Pop-out effects of correct spot during memory-based pursuit

Twenty subjects (10 patients and 10 controls) were tested for this condition to enhance retinal motion inputs of the correct spot as previously described (Ito et al. 2013a; Fukushima et al. 2014). The correct spot remained red in color during the action period (Fig.1A6–7), identical to the fixation spot presented in previous epochs (Fig.1A1–5), but the remaining two spots changed to green with the identical luminance to the correct spot so that the correct spot would stand out as the spot which the subjects must pay attention to, like the fixation spot. Subjects were not told about the color change but were simply asked to perform the task similarly.

### Data analysis

Eye position signals were differentiated by analog circuits (d.c. to 100 Hz, −12 dB/octave) to obtain eye velocity. Visual stimuli, eye position and eye velocity were digitized at 500 Hz by a 16-bit analog/digital board (NB-MIO-16x; National Instruments) using software developed by Fuchs et al. (1994) on a Macintosh computer and were analyzed off-line (Tanaka and Fukushima 1998). Subsequent analyses were performed on Macintosh and Windows computers using homemade (e.g., Fukushima et al. 2000; Shichinohe et al. 2009) and commercial programs (Matlab, MathWorks; Microsoft Excel; KaleidaGraph, Synergy Software). Briefly, all trials during memory-based pursuit were sorted by cue 1/cue 2 direction/instructions (e.g., Fig.1B1–3). Trials in which eyes failed to fixate the stationary spot during the fixation period (Fig.1A1) were omitted. For the remaining traces, typically 55 trials, we calculated correct rates during the action period of go/no-go trials for individual subjects (Fig.1B), then compared correct performance rates after spot motion onset with respect to go/no-go selection and pursuit direction between the two subject groups using the Wilcoxon–Mann–Whitney Test. Since performance of the six patients who received no medication of levodopa/dopamine agonist was similar, their data were combined with those of other patients with Parkinson's disease.

**Figure 2:**
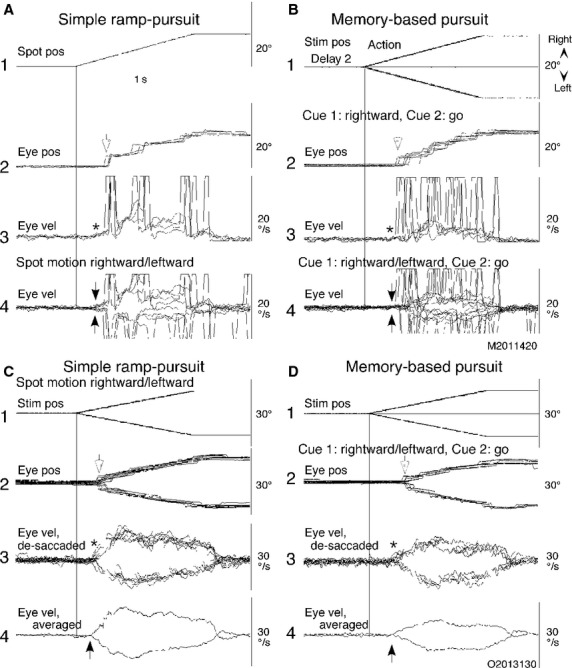
Eye movements of a patient with Parkinson's disease and a control subject. (A and B) eye position and velocity traces of the same patient shown in Figure1B during simple ramp-pursuit (A) and memory-based pursuit (B). (C and D) eye position and desaccaded eye velocity and averaged eye velocity of a control subject aged 85. Rightward and leftward eye movement traces are superimposed in A4, B4 and C2–4, and D2–4 as indicated. In C1, rightward and leftward spot position traces are superimposed. C4 and D4 plot desaccaded and averaged eye velocities for rightward and leftward, separately, during simple ramp-pursuit (C4) and memory-based pursuit (D4) with linear interpolation of the desaccaded portions. See text for further explanation.

Eye movements during the action period of go trials without including error trials and those during simple ramp-pursuit were further analyzed by aligning each trace with spot motion onset (Fig.2). As shown below, one of the 25 patients exhibited high errors during memory-based pursuit (Table1, Pt #25). Therefore, we performed further analysis during go trials in the remaining 24 patients (Table1, Pt #1–24). Traces in which saccades and/or blinks appeared within 100 ms of spot motion onset were omitted. Remaining traces, typically 10–20, were separately averaged for rightward and leftward. In these trials, all subjects fixated the stationary spot well within 100 ms of spot motion onset.

We measured the following parameters as previously described for normal subjects (Ito et al. 2013a; Fukushima et al. 2014): initial pursuit latencies, first saccade latencies, pursuit eye velocities immediately before first saccades, pursuit eye velocities after the first saccades, peak pursuit velocities during pursuit maintenance, and time periods after pursuit onset to the peak velocities. We compared each parameter in patients and controls using t-tests to compare means (significance level corrected for multiple comparisons), as there were unequal numbers of each group (24 patients vs. 14 controls).

Unlike controls, most patients did not exhibit initial pursuit before first saccades during memory-based pursuit (e.g., Fig.2B4 vs. D3–4). Therefore, we first calculated percentage of subjects who exhibited initial pursuit in the memory-based and simple ramp pursuit tasks. For this, we first calculated mean eye velocity for the 100 ms interval immediately before spot motion onset for each subject. Mean values were 0.15 ± 0.05 deg/s for patients and 0.11 ± 0.06 for the controls. If averaged eye velocity before the first correction saccades were within the mean + SD of these control values (i.e., typically <0.2 deg/s), we interpreted that that subject exhibited no initial pursuit. We, then, compared percentages of initial pursuit appearance between the control group and patient group, and also between the two tasks using the chi-square test. UPDRS and FAB scores were also compared between patients who did and those who did not exhibit initial pursuit in the memory task. For those patients who exhibited (6/24 for memory-based pursuit, 19/23 for simple ramp-pursuit, see above), we measured its latencies. During memory-based pursuit, four of the six patients exhibited initial pursuit in both leftward and rightward, and during simple ramp-pursuit 18 of the 19 exhibited initial pursuit in both directions (e.g., Fig.2A4). Pursuit latencies were measured as the time at which mean oppositely directed eye velocities diverged (e.g., Fig.2C4, arrows).

The remaining two patients exhibited initial pursuit only in one direction during memory-based pursuit, and one of the two exhibited initial pursuit only in one direction during simple ramp-pursuit. For those records, we drew two lines on the computer monitor; one along the mean eye velocity before initial pursuit and the other along the initial slope of pursuit eye velocity. The onset of initial pursuit was determined as the time at which the two lines intersected (Carl and Gellman 1987). Since control and most subjects with Parkinson's disease except for the two described above as a whole exhibited little difference in eye movement parameters during pursuit between rightward and leftward in either of the two tasks, data were combined for both directions in each task. Initial pursuit latencies of the patients were averaged by adding values of the two patients who showed initial pursuit only in one direction.

First saccade latencies were calculated by comparing eye position and un-edited eye velocity traces, then, the values were averaged for each subject. Initial pursuit velocities and accelerations of patients that did not exhibit initial pursuit were calculated as zero. To estimate initial pursuit acceleration, pursuit eye velocity immediately before the first saccades was divided by the time difference after pursuit onset. To evaluate postsaccadic enhancement of pursuit eye velocity (Lisberger 1998), we compared pursuit eye velocities before and after first saccades and calculated the difference (mean eye velocity after first saccades – mean eye velocity before the saccades, Fukushima et al. 2014). Peak pursuit eye velocity during pursuit maintenance was measured as the peak desaccaded and averaged eye velocity.

In 20 subjects (10 patients and 10 controls), we compared the time course of pursuit eye velocities during the following three task conditions: simple ramp-pursuit, memory-based pursuit, and memory-based pursuit with popped out correct spot motion. We used ANOVA to test the effects of two factors, subjects (patients vs. controls) and stimulus type (simple ramp-pursuit, memory-based pursuit, and popout), on the measured eye movement parameters. Initial eye acceleration was also calculated by linear regression over the initial 100 ms after pursuit initiation for all tasks as described previously (Ito et al. 2013a; Fukushima et al. 2014).

## Results

### Correct performance rates during memory-based pursuit

Representative eye movements of a patient are illustrated in Figure1B. Sorting all trials by cue 1/cue 2 directions/instructions revealed that she performed all trials correctly with respect to go/no-go selection and pursuit direction after spot motion onset (Fig.1B1–3). Mean ± SD correct rates of the 25 patients and 14 controls were 95.7 ± 9.4 and 98.8 ± 2.0%, respectively. The difference was insignificant between the two groups (median 97.9, and 100%, respectively, *P* = 0.11, Wilcoxon–Mann–Whitney Test). However, one patient (Table1, Pt #25) exhibited an extremely low correct rate (52.6%). Her errors included both go/no-go selection errors and direction errors, consistent with her low FAB score, whereas correct rates of the remaining 24 patients ranged from 90 to 100% with the mean of 97.5 ± 3.0 SD %. As this patient's performance was clearly different, further analysis during go trials was carried out in the remaining 24 patients (Table1, Pt #1–24).

### Eye movement responses during go trials of memory-based pursuit: comparison with simple ramp-pursuit

As described in the Introduction, in normal subjects initial pursuit during the memory-based pursuit task probably arises as a result of extra-retinal activity (e.g., Ito et al. 2013a). Figure2(A and B) illustrates eye movements of the same patient shown in Figure1B during go trials of memory-based pursuit (Fig.2B) and simple ramp-pursuit (Fig.2A) (note expanded time scale). In Figure2B2–3, fewer representative traces are superimposed than in Figure1B1 to illustrate individual eye movement traces when the correct spot moved rightward. During simple ramp-pursuit (Fig.2A1–3), when a single spot moved rightward with the identical trajectory to the correct spot motion in Figure2B2–3, initial pursuit (Fig.2A3, *) was followed by correction saccades (Fig.2A2, arrow), and subsequently followed by smooth-pursuit in which pursuit eye velocities were enhanced in some trials (i.e., postsaccadic enhancement, Lisberger 1998; Fig.2A3). The appearance of initial pursuit before the first correction saccade during simple ramp-pursuit (Fig.2A3, *) is clearly seen by superimposing rightward and leftward eye velocity that followed spot motion in each direction (Fig.2A4, arrows).

Conversely, during memory-based pursuit (Fig.2B1–4), no clear initial pursuit is seen (Fig.2B3, *, B4, arrows) before the first saccades (Fig.2B2, arrow). Thus, during memory-based pursuit, the patient started tracking the correct spot by saccades. Only after saccades did smooth-pursuit eye velocity become clear (Fig.2B4), resulting in prolongation of smooth-pursuit latency.

For comparison, Figure2C and D shows example eye movements of a control subject aged 85 during the identical tasks. During both tasks, initial pursuit clearly appeared (Fig.2C3, D3, *) followed by correction saccades (Fig.2C2, D2, arrows), further followed by enhanced pursuit eye velocity (Fig.2C3, D3). The lack of initial pursuit during the memory-based pursuit task (Fig.2B4, arrows) suggests a qualitatively different response not only between the subject groups (patients vs. controls) but also between the tasks in the same patient (memory-based pursuit vs. simple ramp-pursuit, see below).

### Movement parameters

#### Appearance of initial pursuit

**Figure 3:**
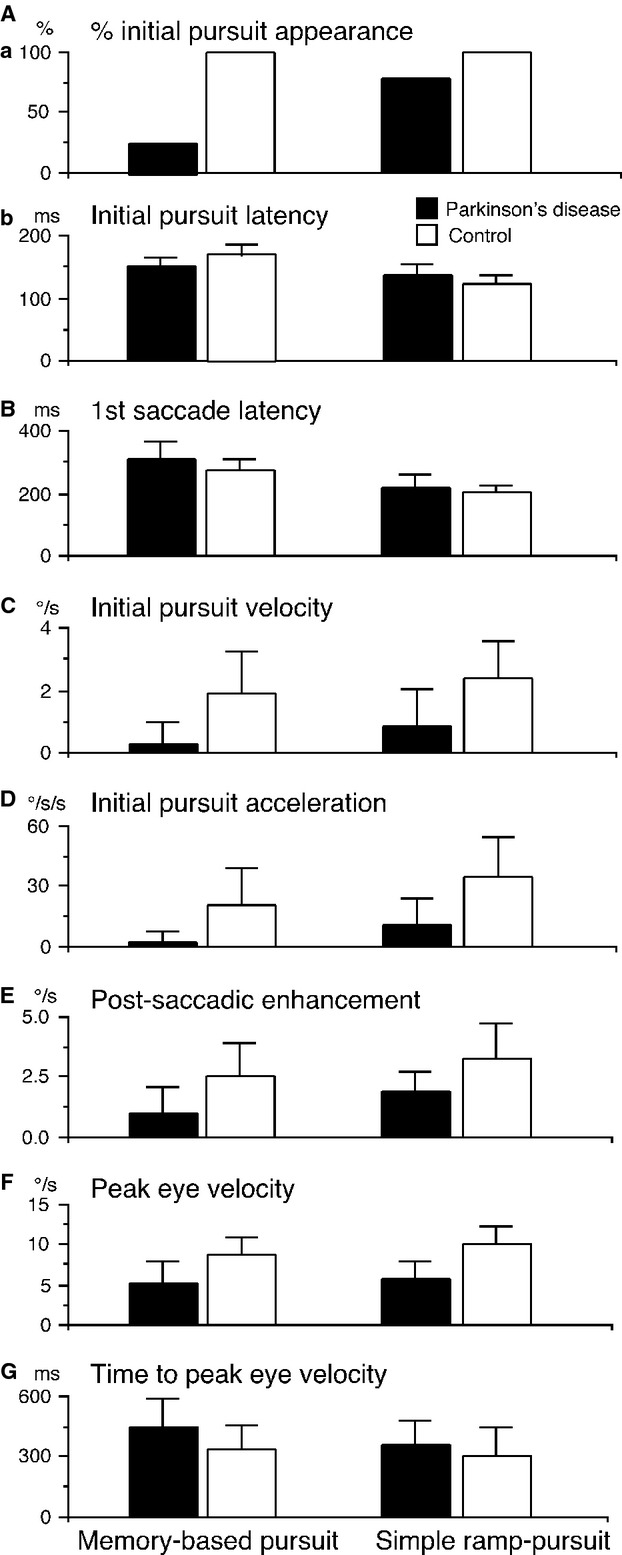
Comparison of movement parameters of patients with Parkinson's disease and control subjects during memory-based pursuit and simple ramp-pursuit. Aa plots % of initial pursuit appearance in each subject group in each task as indicated. Ab-G plot mean + SD values of different movement parameters (ordinates) for patients with Parkinson's disease (solid) and control subjects (open) in each task as indicated. Number of patients tested during memory-based pursuit and simple ramp-pursuit are 24 and 23 in Aa, 6 and 19 in Ab, 24 and 23 in B–G. Number of controls tested are 14 during each task in A–G. See text for further explanation.

Percentages of initial pursuit appearance during memory-based pursuit were different between patients with Parkinson's disease and control subjects (6/24 = 25.0% vs. 14/14 = 100%, *P* < 0.001, chi-square test), and between the two tasks in the patients (6/24 = 25.0% for memory-based pursuit vs. 19/23 = 82.6% for simple ramp-pursuit, *P* < 0.001, chi-square test), whereas all controls tested exhibited initial pursuit in both tasks (14/14, Fig.3Aa). Presence or absence of initial pursuit during the memory task was unrelated to Hoehn–Yahr stages (Table1, Initial pursuit, MP; 1/1 stage 1, 1/4 stage 2–2.5, 4/19 stage ≥ 3).

Also, there was no clear correlation between UPDRS scores and presence or absence of initial pursuit during the memory task. For example, mean ± SD UPDRS scores of the six patients who exhibited initial pursuit and those of the remaining 17 patients who lacked initial pursuit (Table1) were 21.2 ± 7.7 and 25.4 ± 12.2, respectively (median 18.5 and 28.0, respectively, *P* = 0.441, Wilcoxon–Mann–Whitney Test). FAB scores of patients with initial pursuit (*n* = 6) tended to be higher than those of other patients without initial pursuit (*n* = 18) during memory-based pursuit (Table1, median 16.5 and 15.0, respectively, *P* = 0.055, Wilcoxon–Mann–Whitney Test).

#### Latencies of initial pursuit

For the six patients who exhibited initial pursuit during the memory task, their latencies were mostly within the control ranges (see below). Mean ± SD latencies during memory-based pursuit and simple ramp-pursuit were 156.0 ± 15.6 and 135.4 ± 20.3 ms, respectively, in the patients, compared with 177.3 ± 19.6 and 126.3 ± 16.2 ms in the controls (Fig.3Ab).

**Figure 4:**
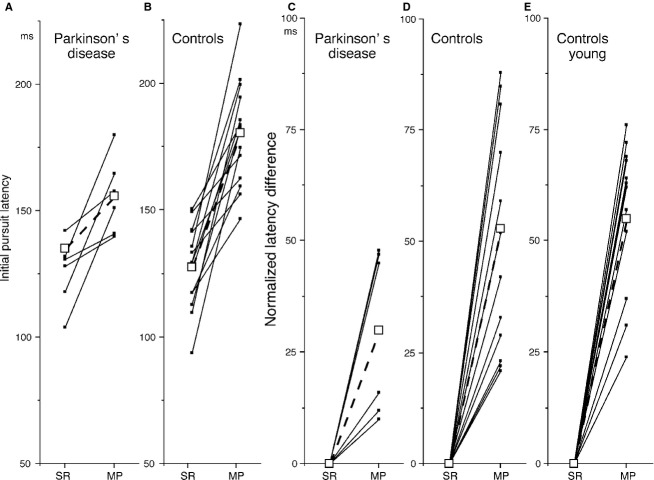
Initial pursuit latencies of individual subjects during simple ramp-pursuit (SR) and memory-based pursuit (MP) and normalized latency differences. (A and B) initial pursuit latencies (from spot motion onset) of six patients who showed initial pursuit during memory-based pursuit (A) and 14 age-matched controls (B). C–D, normalized latency difference (MP–SR) of the six patients (C) and 14 age-matched control subjects (D). For comparison, (E) shows normalized latency difference of young subjects, data taken from Ito et al. (2013a). Values of the same subjects are connected by lines. Open squares connected with dashed lines indicate means.

To further compare latencies of individual subjects who showed initial pursuit in both tasks, Figure4A and B plots their latencies. Latencies during memory-based pursuit were consistently longer than those of simple ramp-pursuit in individual subjects. Normalized latency differences (memory-based pursuit – simple ramp-pursuit) are plotted for the six patients (Fig.4C) and 14 controls (D). Mean ± SD differences in latencies between the two tasks were 30.0 ± 19.1 in the patients and 52.5 ± 24.2 ms in controls (Fig.4C and D, open squares). The distribution between the two groups (Fig.4C vs. D) is clearly different (see Discussion).

#### Latencies of first correction saccades

Latencies of the first saccades were significantly longer in the patients than controls in both the simple ramp (*P* = 0.016) and memory pursuit (*P* < 0.001) tasks. In both groups of subjects, their latencies were longer during memory-based pursuit than for simple ramp-pursuit. Mean ± SD latencies of the patients during the two tasks were 310.2 ± 56.7 and 220.0 ± 35.8 ms compared with 273.6 ± 32.4 and 202.6 ± 24.7 ms for the controls (Fig.3B).

#### Initial pursuit eye velocity

Initial pursuit velocities before the first saccades were significantly lower in the patients than controls in both the simple ramp (*P* < 0.001) and memory pursuit (*P* < 0.001) tasks and in both groups their mean velocities were lower during memory-based pursuit than for simple ramp-pursuit. Mean±SD velocities of the patients during memory-based pursuit and simple ramp-pursuit were 0.3 ± 0.6 and 0.9 ± 0.7°/s, whereas those of the controls were 1.9 ± 1.3 and 2.4 ± 1.2 ^o^/s (Fig.3C).

#### Initial pursuit acceleration

Initial pursuit accelerations were also significantly lower in the patients than the controls in both the simple ramp (*P* < 0.001) and memory pursuit (*P* < 0.001) tasks, and in both groups their mean accelerations were lower during memory-based pursuit than for simple ramp pursuit. Mean ± SD values of the patients during the two tasks were 2.1 ± 4.9 and 11.4 ± 12.2°/s/s compared with 20.7 ± 18.5 and 34.5 ± 20.3°/s/s in the controls (Fig.3D).

#### Postsaccadic enhancement of pursuit eye velocity

Postsaccadic enhancement (Lisberger 1998) was significantly weaker in the patients in both the simple ramp (*P* < 0.001) and memory pursuit (*P* < 0.001) tasks. Mean ± SD differences (pursuit velocities after saccades – before saccades) of the patients during memory-based pursuit and simple ramp-pursuit were 1.0 ± 1.1 and 1.8 ± 0.8°/s compared with 2.5 ± 1.4 and 3.3 ± 1.4 ^o^/s in the controls (Fig.3E).

#### Peak pursuit eye velocity during pursuit maintenance

Peak pursuit eye velocity was significantly lower in the patients in both the simple ramp (*P* < 0.001) and memory pursuit (*P* < 0.001) tasks. Mean ± SD values of the patients during memory-based pursuit and simple ramp-pursuit were 5.3 ± 2.6 and 5.9 ± 2.0°/s compared with 8.8 ± 2.3 and 10.2 ± 2.0 ^o^/s in the controls (Fig.3F).

#### Time to peak pursuit eye velocity

Time periods to reach peak pursuit eye velocities after pursuit onset were significantly longer in the patients than controls during memory-based pursuit (*P* = 0.003), but not during simple ramp pursuit (*P* = 0.17). Mean ± SD values of the patients during memory-based pursuit and simple ramp-pursuit were 444.1 ± 147.2 and 359.1 ± 117.4 ms compared with 341.0 ± 132.9 and 313.3 ± 147.7 ms in the controls (Fig.3G).

### Pursuit eye velocity time courses and pop-out effects of correct spot during memory-based pursuit

To further examine whether cue-information priming was deficient in Parkinson's disease, we tested pop-out effects of the correct spot (see Materials and Methods). If patients with Parkinson's disease indeed have deficient priming ability, they would have difficulty inducing response enhancement to the popped-out spot.

**Figure 5:**
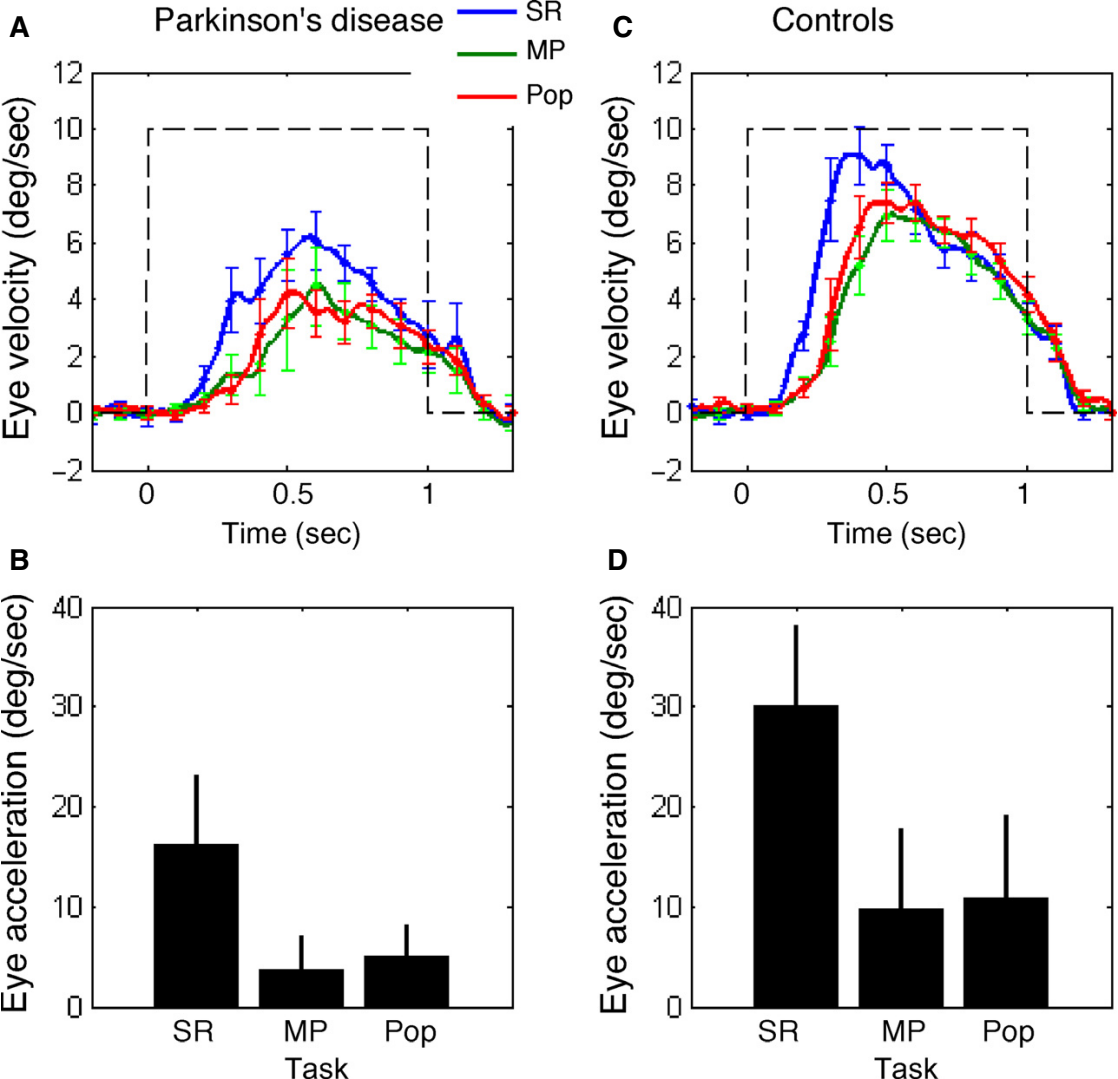
Comparison of eye velocity time courses of patients with Parkinson's disease and normal controls. (A and C) blue, green, and red traces are mean ± SEM desaccaded eye velocity of 10 patients (A) and 10 controls (C) during simple ramp-pursuit (SR), memory-based pursuit (MP) and popping-out of the correct spot during memory-based pursuit (Pop). (B and D) pursuit acceleration of the 10 patients (B) and 10 controls (D) during the three task conditions for the 1st 100 m after response onset. Error bars indicate one SEM. For further explanation, see text.

To compare eye velocity time courses of the three task conditions, Figure5A and C plots mean ± SEM eye velocities of the patients (A) and control subjects (C) during simple ramp-pursuit (blue, SR), memory-based pursuit (green, MP), and popping-out of the correct spot (red, Popout). Eye velocity time courses during the three conditions were qualitatively similar in the two subject groups, but, in all task conditions, responses of the patients were smaller than those of control subjects. Popout raised the mean peak eye velocity above the MP condition in controls, but not in the patients. Two factor analysis of variance used to compare eye velocities at 50 ms intervals from 100 to 400 ms after target onset indicated that there were significant effects of both subject group and test condition (SR, MP, Popout) from 150 ms onward. The peak difference between SR and MP occurred at 350 ms, at which time there was a significant effect of group (*F*_1,54_ = 23.06; *P* < 0.001) and test condition (*F*_2,54_ = 13.55; *P* < 0.001). Multiple comparisons (with Bonferroni correction) showed that there was always a significant difference between SR and MP and between SR and Popout at 350 ms, but in neither group was there any significant difference between MP and Popout despite the increase in mean peak velocity in the controls.

Figure5B and D compares initial eye acceleration of the same subjects obtained by linear regression over the initial 100 ms after pursuit initiation in all test conditions (see Data analysis). Initial eye acceleration (1st 100 ms) was lower during both memory-based pursuit and the pop-out condition than during simple ramp-pursuit in both subject groups.

## Discussion

### Nature of impaired pursuit in Parkinson's disease during the memory-based pursuit task

By separating two major components of prediction during memory-based pursuit (see Introduction), our results show that cue-information memory was normal in most patients with Parkinson's disease (24/25 = 96%), but movement preparation and execution were impaired. In particular, most of them (18/24 = 75%) lacked initial pursuit during the memory-based (but not simple ramp) pursuit task, indicating specific dysfunction of pursuit initiation mechanisms based on extra-retinal information, as discussed below.

### Cue-information memory during the memory-based pursuit task in Parkinson's disease

Our results showed that there was no significant difference in correct rates between the patient group (*n* = 25) and the age-matched control group (*n* = 14), indicating that cue-information memory during the memory task was normal. As the results were similar with and without medication of the levodopa/dopamine agonists (Table1), normal cue-information memory in this task was not due to the medication. However, as one patient (Table1, Pt #25) clearly exhibited impaired cue-information memory despite similar clinical findings including normal brain MRI, and as we were unable to test this task in the five patients who exhibited low FAB scores (see Subjects in Materials and methods, Table1, Pt #26–30), our results indicate that some patients with Parkinson's disease (at least 1/25 = 4%) have deficient cue-information memory in our memory task (cf., Harrington et al. 1990; Possin et al. 2008; Lee et al. 2010). Ladda et al. (2008) reported that the use of visual information as a cue for predictive pursuit affected its latency in patients with mild to moderate Parkinson's disease, and interpreted that the latency prolongation was due to deficient spatial memory (see below for our interpretation).

### Impaired pursuit preparation and priming deficiency in Parkinson's disease during the memory-based pursuit task

In contrast to the consistent appearance of initial pursuit in the cued direction in control subjects tested (14/14 = 100%, Fig.2D), the lack of initial pursuit in most patients (Figs.2B, 3Aa, 18/24 = 75%) and latency prolongation of the first saccades (Fig.3B) in the same task suggest that they have difficulty in inducing priming effects based on the cue-information memory. Even in those patients who exhibited initial pursuit, its velocity and acceleration were significantly lower than those of control subjects (Fig.3C, D). Our results using the popped-out correct spot confirm that patients with Parkinson's disease indeed had deficient priming ability, as the velocity profile of initial pursuit during memory-based pursuit was basically similar even with the presence of popped-out spot (Fig.5A). However, Popout also gave little enhancement in the age-matched controls either. This finding is consistent with results of a previous experiment (Fukushima et al. 2014), in which Popout enhancement had been shown in young controls but not in a more elderly control group. The lack of initial pursuit during the memory task in most patients is in contrast to the appearance of initial pursuit in most of the same patients during simple ramp-pursuit (Fig.3Aa, 19/23 = 83%), indicating the task specificity of this component in Parkinson's disease.

Warabi et al. (2011) compared latencies of rapid movements of eyes (i.e., saccades) and wrist to a visual stimulus during gap and overlap tasks, and showed that the latency prolongation primarily reflected the difficulty in terminating existing movement/posture in Parkinson's disease. In this study, the difficulty in terminating fixation may contribute to the latency prolongation of the first saccades (vs. controls) during the two tasks (Fig.3B).

### Impaired pursuit preparation/execution and deficient extra-retinal drive in Parkinson's disease

Extra-retinal drive contributes to predictive pursuit initiation in normal humans (Barnes and Collins 2008a,b; Helmchen et al. 2012). Ito et al. (2013a) have further shown in normal subjects that during initial blanking of spot motion in the memory-based pursuit task, initial pursuit appears in the correct direction. This extra-retinal predictive component had dynamic characteristics similar to those exhibited by the build-up of pursuit maintenance during target blanking, suggesting participation of a common extra-retinal drive in pursuit maintenance and prediction-related pursuit initiation in normal subjects (Barnes and Collins 2008a,b). Conversely, impairment of both functions in Parkinson's disease (Fig.3) suggests deficient extra-retinal drive during memory-based pursuit. This suggestion was verified by Helmchen et al. (2012) who, using the task devised by Barnes and Collins (2008b), demonstrated that Parkinson's disease patients indeed had difficulties in pursuit initiation when the target was extinguished shortly before motion onset.

Our results showing that most Parkinson's disease patients lacked initial pursuit during the memory-based (not simple ramp) pursuit task (Figs.2B4 vs. D4, 3Aa) are consistent with the common notion that one of the main disorders of Parkinson's disease is the difficulty in initiating internally triggered (in contrast to externally triggered, reflexive) movements (e.g., Bloxham et al. 1984; Crawford et al. 1989; also Helmchen et al. 2012). Our results extend this notion by showing that the lack of initial pursuit was due to specific dysfunction of the pursuit initiation mechanisms based on extra-retinal information including cue-information priming and extra-retinal drive, resulting in prolongation of smooth-pursuit latency, as it appeared after saccades (e.g., Fig.2B4), but not due to dysfunction of cue-information memory (cf., Ladda et al. 2008).

Effects of dopamine treatment on smooth-pursuit of patients with Parkinson's disease are conflicting; Gibson et al. (1987) reported that dopamine treatment was effective (also Rascol et al. 1989; Bareš et al. 2003), whereas Sharpe et al. (1987) reported it was ineffective (also Ladda et al. 2008; Pinkhardt et al. 2012). Although the number of patients without medication was small (Table1, no drug), our results seem to be consistent with the results reported by Sharpe et al. (1987; also Ladda et al. 2008; Pinkhardt et al. 2012).

Impaired control of saccades occurs in Parkinson's disease (e.g., Cameron et al. 2010). In our study, some patients exhibited saccades during delay 2 before the action period (e.g., Fig.1B1). As their performance was mostly corrected by verbal feedback (see Materials and Methods), we interpret such premature saccades in our task as their strategy of compensating for the impaired movement parameters (Fig.3, also Warabi et al. 1988), but not the impaired saccade control for the following reason. The minority of patients (6/24) exhibited initial pursuit during memory-based pursuit with the mean latency shorter than that of the control subjects (Fig.3Ab). Further comparison of the distribution of normalized latency difference between memory-based pursuit and simple ramp-pursuit (Fig.4C–E) indicates that, unlike the distribution of age-matched controls (Fig.4D) and young controls in the previous study (Fig.4E, Ito et al. 2013a), these six patients (6/24) showed much shorter latency difference (Fig.4C vs. D–E), suggesting their strategy of compensation by setting shorter decision-making delay during memory-based pursuit.

### Neural correlates for the extra-retinal mechanisms to initiate memory-based pursuit

In trained monkeys, initial pursuit during the action period of the memory task depends on normal activity of the supplementary eye fields (SEF) and frontal eye fields (FEF) for the following reasons (Shichinohe et al. 2009; Fukushima et al. 2011a); (1) cue 1 direction memory and cue 2 go instruction enhance visual motion responses of neurons in those areas in the cued direction, and (2) chemical inactivation of these areas impairs initial pursuit. However, chemical inactivation of the two areas resulted in different effects; SEF inactivation did not impair pursuit maintenance, but resulted in significantly higher direction errors and go/no-go selection errors (Shichinohe et al. 2009). FEF inactivation, in contrast, did not induce such errors but decreased pursuit eye velocity during pursuit maintenance, resulting in “saccadic tracking” (Fukushima et al. 2011a; Mahaffy and Krauzlis 2011). These results indicate that, although both areas are involved in smooth-pursuit prediction, the SEF is primarily involved in planning based on cue-information memory, whereas the FEF is primarily involved in generating motor commands for efficient pursuit execution (also Yang and Heinen 2014). Involvement of FEF pursuit neurons in extra-retinal pursuit components has been shown using a single spot (Tanaka and Fukushima 1998; Fukushima et al. 2000, 2002) and during memory-based pursuit (Fukushima et al. 2011a).

### Possible pathophysiology of impaired pursuit in Parkinson's disease

Involvement of the basal ganglia in automatic movements has been suggested (for reviews, see Marsden 1982; Redgrave et al. 2010). Cui et al. (2003) reported projection of the FEF pursuit area to the basal ganglia in monkeys, output of which further projects back to the FEF through the thalamus, thus forming a possible pursuit loop between the FEF and basal ganglia (Lynch and Tian 2006). Confirmation of pursuit signals in the globus pallidus (Yoshida and Tanaka 2009) and central thalamus (Tanaka 2005) supports their proposal (Cui et al. 2003; Lynch 2009).

As the extra-retinal mechanisms including cue-information priming and extra-retinal drive were specifically impaired in most patients with Parkinson's disease during memory-based pursuit as discussed above, the basal ganglia, especially, the FEF/basal ganglia loop (Lynch and Tian 2006) may be involved in these mechanisms for efficient memory-based pursuit initiation, and its dysfunction may result in deficient pursuit commands from the FEF to the brainstem (Fukushima et al. 2011c, 2013).

In contrast to the normal cue-information memory in most patients with Parkinson's disease during memory-based pursuit, significantly higher error rates were observed in patients with frontal cortical dysfunction (Ito et al. 2011) and progressive supranuclear palsy (PSP, Ito et al. 2013b), indicating dysfunction of cue-information memory in those patients. Taken together, our results suggest clinical usefulness of our tasks. As chemical inactivation of the SEF or FEF in monkeys resulted in different effects, in particular, SEF inactivation resulted in significantly higher errors during memory-based pursuit as described above (Shichinohe et al. 2009), these results taken together suggest that Parkinson's disease patients with impaired cue-information memory (e.g., Table1, Pt #25) and most PSP patients may have frontal cortical dysfunction that includes the SEF (cf., Possin et al. 2008; Lee et al. 2010). Projections of the basal ganglia to the SEF through the thalamus are also known (Parthasarathy et al. 1992; see Tanji 1994 for review).

## Conclusions

By comparing tracking eye movement in tasks that did or did not require cue-information memory, most patients with Parkinson's disease exhibited normal cue-information memory, but movement preparation and execution were impaired. In particular, most of them lacked initial pursuit during the memory-based (but not simple ramp) pursuit task, indicating specific dysfunction of pursuit initiation mechanisms based on extra-retinal information, including cue-information priming and extra-retinal drive. Further comparison with studies in trained monkeys suggested a dysfunctional pursuit loop between the FEF and basal ganglia.

## References

[b1] Barborica A, Ferrera VP (2003). Estimating invisible target speed from neuronal activity in monkey frontal eye field. Nat. Neurosci.

[b2] Bareš M, Brázdila M, KaňovskÝ P, Jurák P, Daniela P, Kukletac M (2003). The effect of apomorphine administration on smooth pursuit ocular movements in early parkinsonian patients. Parkinsonism Relat. Disord.

[b3] Barnes GR (2008). Cognitive processes involved in smooth pursuit eye movements. [Review]. Brain Cogn.

[b4] Barnes GR, Collins CJS (2008a). The influence of briefly presented randomised target motion on the extra-retinal component of ocular pursuit. J. Neurophysiol.

[b5] Barnes GR, Collins CJS (2008b). Evidence for a link between the extra-retinal component of random-onset pursuit and the anticipatory pursuit of predictable object motion. J. Neurophysiol.

[b6] Becker W, Fuchs AF (1985). Prediction in the oculomotor system: smooth pursuit during transient disappearance of a visual target. Exp. Brain Res.

[b7] Bichot NP, Schall JD (2002). Priming in macaque frontal cortex during popout visual search: feature-based facilitation and location-based inhibition of return. J. Neurosci.

[b8] Bloxham CA, Mindel TA, Frith CD (1984). Initiation and execution of predictable and unpredictable movements in Parkinson's disease. Brain.

[b9] Bronstein AM, Kennard C (1985). Predictive ocular motor control in Parkinson's disease. Brain.

[b10] Cameron IJM, Watanabe M, Pari G, Munoz DP (2010). Executive impairment in Parkinson's disease: response automaticity and task switching. Neuropsychol.

[b11] Carl JR, Gellman RS (1987). Human smooth pursuit: stimulus-dependent responses. J. Neurophysiol.

[b12] Crawford TJ, Henderson L, Kennard C (1989). Abnormalities of nonvisually-guided eye movements in Parkinson's disease. Brain.

[b13] Cui DM, Yan YJ, Lynch JC (2003). Pursuit subregion of frontal eye field projects to the caudate nucleus in monkeys. J. Neurophysiol.

[b14] Dubois B, Slachevsky A, Livtan I, Pillon B (2000). The FAB. A frontal assessment battery at bedside. Neurology.

[b15] Fahn S, Fahn S, Marsden CD, Goldstein M, Calne DB, Elton RL, UPDRS program members (1987). Unified Parkinsons Disease Rating Scale. Recent developments in Parkinson's disease.

[b16] Flowers KA, Downing AC (1978). Predictive control of eye movements in Parkinson disease. Ann. Neurol.

[b17] Folstein MF, Folstein SE, McHugh PR (1975). ‘‘Mini-mental state’’. A practical method for grading the cognitive state of patients for the clinician. J. Psych. Res.

[b18] Fuchs AF, Robinson FR, Straube A (1994). Participation of the caudal fastigial nucleus in smooth-pursuit eye movements. I. Neuronal activity. J. Neurophysiol.

[b19] Fukushima K, Sato T, Fukushima J, Shinmei Y, Kaneko CRS (2000). Activity of smooth pursuit-related neurons in the monkey periarcuate cortex during pursuit and passive whole body rotation. J. Neurophysiol.

[b20] Fukushima K, Yamanobe T, Shinmei Y, Fukushima J (2002). Predictive responses of peri-arcuate pursuit neurons to visual target motion. Exp. Brain Res.

[b21] Fukushima K, Akao T, Shichinohe N, Nitta T, Kurkin S, Fukushima J (2008). Predictive signals in the pursuit area of the monkey frontal eye fields. Prog. Brain Res.

[b22] Fukushima J, Akao T, Shichinohe N, Kurkin S, Kaneko CRS, Fukushima K (2011a). Neuronal activity in the caudal frontal eye fields of monkeys during memory-based smooth-pursuit eye movements: comparison with the supplementary eye fields. Cereb. Cor.

[b23] Fukushima K, Fukushima J, Kaneko CRS, Belton T, Ito N, Olley PM (2011b). Memory-based smooth-pursuit: neuronal mechanisms and preliminary results of clinical application. Ann. N. Y. Acad. Sci.

[b24] Fukushima K, Fukushima J, Warabi T (2011c). Vestibular-related frontal cortical areas and their roles in smooth-pursuit eye movements: representation of neck velocity, neck-vestibular interactions and memory-based smooth-pursuit. Front. Neurol.

[b25] Fukushima K, Fukushima J, Warabi T, Barnes GR (2013). Cognitive processes involved in smooth pursuit eye movements: behavioral evidence, neural substrate and clinical correlation. Front. Sys. Neurosci.

[b26] Fukushima K, Barnes GR, Ito N, Olley PM, Warabi T (2014). Normal aging affects movement execution but not visual motion working memory and decision-making delay during cue-dependent memory-based smooth-pursuit. Exp. Brain Res.

[b27] Garbutt S, Lisberger SG (2006). Directional cuing of target choice in human smooth pursuit eye movements. J. Neurosci.

[b28] Gibson JM, Pimlott R, Kennard C (1987). Ocular motor and manual tracking in Parkinson's disease and the effect of treatment. J. Neurol. Neurosurg. Psychiatr.

[b29] Harrington DL, Haaland KY, Yeo RA, Marder E (1990). Procedural memory in Parkinson's disease: impaired motor but not visuoperceptual learning. J. Clin. Exp. Neuropsychol.

[b30] Helmchen CH, Pohlmann J, Trillenberg P, Lencer R, Graf J, Sprenger A (2012). Role of anticipation and prediction in smooth pursuit eye movement. Mov. Disord.

[b31] Hoehn MM, Yahr MD (1967). Parkinsonism onset, progression, and mortality. Neurology.

[b32] Hughes AJ, Daniels SE, Kilford L, Lees AJ (1992). Accuracy of clinical diagnosis of idiopathic Parkinson's disease: clinico-pathological study of 100 cases. J. Neurol. Neurosurg. Psychiatr.

[b33] Ito N, Ikeno K, Kobayashi N, Takei H, Olley PM, Chiba S (2011). Clinical application of a memory- based smooth pursuit eye movement (SPEM) task to patients with idiopathic Parkinson's disease (PD) and patients with frontal dysfunction. [Abstract]. Neurosci. Res.

[b34] Ito N, Tamaki N, Masuno A, Ikeno K, Onishi S, Kobayashi N (2012).

[b35] Ito N, Barnes GR, Fukushima J, Fukushima K, Warabi T (2013a). Cue-dependent memory-based smooth-pursuit in normal human subjects: importance of extra-retinal mechanisms for initial pursuit. Exp. Brain Res.

[b36] Ito N, Tamaki N, Masuno A, Ikeno K, Onishi S, Kobayashi N (2013b).

[b37] Kurkin S, Akao T, Shichinohe N, Fukushima J, Fukushima K (2011). Neuronal activity in Medial Superior Temporal area (MST) during memory-based smooth-pursuit eye movements in monkeys. Exp. Bain Res.

[b38] Kurkin S, Akao T, Fukushima J, Shichinohe N, Kaneko CRS, Belton T (2014). *No-go* neurons in the cerebellar oculomotor vermis and caudal fastigial nuclei: planning tracking eye movements. Exp. Brain Res.

[b39] Ladda J, Valkovič P, Eggert T, Straube A (2008). Parkinsonian patients show impaired predictive smooth pursuit. J. Neurol.

[b40] Lee E-Y, Cowan N, Vogel EK, Rolan T, Valle-Inclán F, Hackley SA (2010). Visual working memory deficits in patients with Parkinson's disease are due to both reduced storage capacity and impaired ability to filter out irrelevant information. Brain.

[b41] Leigh R, Zee DS (2006). The neurology of eye movements.

[b42] Lekwuwa GU, Barnes GR, Collins CJS, Limousin P (1999). Progressive bradykinesia and hypokinesia of ocular pursuit in Parkinson's disease. J. Neurol. Neurosurg. Psychiatr.

[b43] Leventhal AG, Wang YC, Pu ML, Zhou YF, Ma YY (2003). GABA and its agonists improved visual cortical function in senescent monkeys. Science.

[b44] Lisberger SG (1998). Postsaccadic enhancement of initiation of smooth pursuit eye movements in monkeys. J. Neurophysiol.

[b45] Lisberger SG, Ferrera VP (1997). Vector averaging for smooth pursuit eye movements initiated by two moving targets in monkeys. J. Neurosci.

[b46] Lynch JC (2009). Pursuit eye movement signals in the basal ganglia. NeuroReport.

[b47] Lynch JC, Tian J-R (2006). Cortico-cortical networks and cortico-subcortical loops for the higher control of eye movements. [Review]. Prog. Brain Res.

[b48] Mahaffy S, Krauzlis RA (2011). Inactivation and stimulation of the frontal pursuit area change pursuit metrics without affecting pursuit target selection. J. Neurophysiol.

[b49] Marsden CD (1982). The mysterious motor functions of the basal ganglia: the Robert Wartenberg Lecture. Neurology.

[b50] Moschner C, Baloh RW (1994). Age-related changes in visual tracking. J. Gerontol.

[b51] Parthasarathy HB, Schall JD, Graybiel AM (1992). Distributed but convergent ordering of corticostriatal projections: analysis of the frontal eye field and the supplementary eye field in the macaque monkey. J. Neurosci.

[b52] Pinkhardt EH, Jürgens R, Becker W, Valdarno F, Ludolph AC, Kassubek J (2008). Differential diagnostic value of eye movement recording in PSP-parkinsonism, Richardson's syndrome, and idiopathic Parkinson's disease. J. Neurol.

[b53] Pinkhardt EH, Kassubek J, Süssmuth S, Ludolph AC, Becker W, Jürgens R (2009). Comparison of smooth pursuit eye movement deficits in multiple system atrophy and Parkinson's disease. J. Neurol.

[b54] Pinkhardt EH, Jürgens R, Lulé D, Heimrath J, Ludolph AC, Becker W (2012). Eye movement impairments in Parkinson's disease: possible role of extradopaminergic mechanisms. BMC Neurol.

[b55] Possin KL, Filoteo JV, Song DD, Salmon DP (2008). Spatial and object working memory deficits in Parkinson's disease are due to impairment in different underlying processes. Neuropsychol.

[b56] Rascol O, Clanet M, Montastruc JL, Simonetta M, Soulier-Esteve MJ, Doyon B (1989). Abnormal ocular movements in Parkinson's disease. Evidence for involvement of dopaminergic systems. Brain.

[b57] Redgrave P, Rodriguez M, Smith Y, Rodriguez-Oroz MC, Lehericy S, Bergman H (2010). Goal-directed and habitual control in the basal ganglia: implications for Parkinson's disease. [Review]. Nat. Rev. Neurosci.

[b58] Rottach KF, Riley DE, DiScenna AO, Zivotofsky AZ, Leigh RJ (1996). Dynamic properties of horizontal and vertical eye movements in parkinsonian syndromes. Ann. Neurol.

[b59] Rowland LP, Pedley TA (2010). Merritt's neurology.

[b60] Sharpe JA, Fletcher WA, Lang AE, Zackon DH (1987). Smooth pursuit during dose-related on-off fluctuations in Parkinson's disease. Neurology.

[b61] Shibasaki H, Tsuji S, Kuroiwa Y (1979). Oculomotor abnormalities in Parkinson's disease. Arch. Neurol.

[b62] Shichinohe N, Akao T, Kurkin S, Fukushima J, Kaneko CRS, Fukushima K (2009). Memory and decision making in the frontal cortex during visual motion processing for smooth pursuit eye movements. Neuron.

[b63] Sprenger A, Trillenberg P, Pohlmann J, Herold K, Lencer R, Helmchen C (2011). The role of prediction and anticipation on age-related effects on smooth pursuit eye movements. Ann. N. Y. Acad. Sci.

[b64] Tanaka M (2005). Involvement of the central thalamus in the control of smooth pursuit eye movements. J. Neurosci.

[b65] Tanaka M, Fukushima K (1998). Neuronal responses related to smooth pursuit eye movements in the periarcuate cortical area of monkeys. J. Neurophysiol.

[b66] Tanji J (1994). The supplementary motor area in the cerebral cortex. [Review]. Neurosci. Res.

[b67] Vidailhet M, Rivaud S, Gouider-Khouja N, Pillon B, Bonnet AM, Gaymard B (1994). Eye movements in parkinsonian syndromes. Ann. Neurol.

[b68] Warabi T, Yanagisawa N, Shindo R (1988). Changes in strategy of aiming tasks in Parkinson's disease. Brain.

[b69] Warabi T, Fukushima K, Olley PM, Chiba S, Yanagisawa N (2011). Difficulty in terminating the preceding movement/posture explains the impaired initiation of new movements in Parkinson's disease. Neurosci. Lett.

[b70] Waterston JA, Barnes GR, Grealy MA, Collins S (1996). Abnormalities of smooth eye and head movement control in Parkinson's disease. Ann. Neurol.

[b71] White OB, Saint-Cyr A, Tomlinson D, Sharpe JA (1983). Ocular motor deficits in Parkinson's disease. II. Control of the saccadic and smooth pursuit systems. Brain.

[b72] Yang S, Heinen S (2014). Contrasting the roles of the supplementary and frontal eye fields in ocular decision making. J. Neurophysiol.

[b73] Yoshida A, Tanaka M (2009). Neuronal activity in the primate globus pallidus during smooth pursuit eye movements. NeuroReport.

